# Clinical decision support systems for improving diagnostic accuracy and achieving precision medicine

**DOI:** 10.1186/s13336-015-0019-3

**Published:** 2015-03-26

**Authors:** Christian Castaneda, Kip Nalley, Ciaran Mannion, Pritish Bhattacharyya, Patrick Blake, Andrew Pecora, Andre Goy, K Stephen Suh

**Affiliations:** Genomics and Biomarkers Program, Hackensack University Medical Center, Hackensack, NJ 07601 USA; Sophic Alliance, 2275 Research Blvd., Suite 500, Rockville, MD 20850 USA; Department of Pathology, Hackensack University Medical Center, Hackensack, NJ 07601 USA; John Theurer Cancer Center, Hackensack University Medical Center, D. Jurist Research Building, 40 Prospect Avenue, Hackensack, NJ 07601 USA

**Keywords:** Personalized medicine, Precision medicine, Bioinformatics, Clinical informatics, Clinical decision support system, Clinical outcome, Patient care, Integrated knowledge environment, Artificial intelligence, Watson

## Abstract

As research laboratories and clinics collaborate to achieve precision medicine, both communities are required to understand mandated electronic health/medical record (EHR/EMR) initiatives that will be fully implemented in all clinics in the United States by 2015. Stakeholders will need to evaluate current record keeping practices and optimize and standardize methodologies to capture nearly all information in digital format. Collaborative efforts from academic and industry sectors are crucial to achieving higher efficacy in patient care while minimizing costs. Currently existing digitized data and information are present in multiple formats and are largely unstructured. In the absence of a universally accepted management system, departments and institutions continue to generate silos of information. As a result, invaluable and newly discovered knowledge is difficult to access. To accelerate biomedical research and reduce healthcare costs, clinical and bioinformatics systems must employ common data elements to create structured annotation forms enabling laboratories and clinics to capture sharable data in real time. Conversion of these datasets to knowable information should be a routine institutionalized process. New scientific knowledge and clinical discoveries can be shared via integrated knowledge environments defined by flexible data models and extensive use of standards, ontologies, vocabularies, and thesauri. In the clinical setting, aggregated knowledge must be displayed in user-friendly formats so that physicians, non-technical laboratory personnel, nurses, data/research coordinators, and end-users can enter data, access information, and understand the output. The effort to connect astronomical numbers of data points, including ‘-omics’-based molecular data, individual genome sequences, experimental data, patient clinical phenotypes, and follow-up data is a monumental task. Roadblocks to this vision of integration and interoperability include ethical, legal, and logistical concerns. Ensuring data security and protection of patient rights while simultaneously facilitating standardization is paramount to maintaining public support. The capabilities of supercomputing need to be applied strategically. A standardized, methodological implementation must be applied to developed artificial intelligence systems with the ability to integrate data and information into clinically relevant knowledge. Ultimately, the integration of bioinformatics and clinical data in a clinical decision support system promises precision medicine and cost effective and personalized patient care.

## Introduction

*Clinical informatics* is the application of informatics and information technology to support healthcare delivery services. Its role is rapidly evolving toward providing better clinical decision-making by integrating state-of-the-art knowledge with medical record systems [1]. As medicine moves into an era of personalized treatment and precision pharmaceuticals, the application of expertise in electronic health/medical record (EHR/EMR) systems and translational research will enhance operating efficiencies for hospitals and reduce costs. In reality, the populating and analyzing of large amounts of accumulating data in standardized format from EHRs has yet to happen, since protocols and resources have not yet sufficiently matured. Recognition of the importance of applying digitized data and information for patient care has spurred the first class of physicians to become board-certified in the newly-created subspecialty of clinical informatics [2].

*Bioinformatics* is the development of storage, analytic, and interpretive methods to optimize the transformation of increasingly voluminous biomedical and genomic data into proactive, predictive, preventive, and participatory healthcare [3]. The field of bioinformatics has grown exponentially, providing a vast amount of information, information that is largely unstandardized and practically inaccessible. While problems with semantic medical term standardization and varied data quality plague clinical informatics, research scientists continue to struggle with different exchange data types, service ontologies, and fragmented web services [4,5]. As we move into the era of the $1,000 genome analysis [6] and the reality of mandatory EHR [7], the focus of bioinformatics has shifted from gathering data to analyzing the massive amounts of available data for direct application in patient care.

A critical step for achieving precision medicine will be to integrate old and new data into validated information and to convert this information into knowledge directly applicable to diagnosis, prognosis, or treatment. This will entail developing an integrated knowledge environment that continually captures information, grows, accumulates, organizes, and institutionalizes new information, making it accessible to health care providers. Knowledge accumulated from scientific research and clinical data contained in EHRs will be shared and will impact the discovery of novel therapeutic methods and the application of precision medicine. It now takes 17 years for a laboratory discovery to reach widespread clinical application [8]. The promise of bioinformatics is to optimize treatment decisions and enhance outcomes, by providing immediate access to the patient’s genomic, laboratory, and EHR information, and by relating that information to current clinical trials and research.

## Role of EHR in linking clinical informatics and bench science

The advantages and disadvantages of EHR have been debated since passage of the American Recovery and Reinvestment and the HITECH Acts of 2009 mandated and incentivized their adoption and use [9]. Although analysis by the American Medical College of Informatics have outlined cost, data security concerns, and steep learning curves as the major barriers to EHR adoption [10], a majority of health care providers perceive that implementation and meaningful use of EHR will decrease operating costs, decrease error rates, and increase favorable patient outcomes [11]. From an informatics perspective, EHR implementation will permit creation of centralized locations for clinical data, assay results, and patient outcomes, which will benefit biomedical research. Proactive use of EHR will modernize patient care in all sectors of medicine [12]. As described by Blake et al., for example, an EHR database featuring a universal, user-friendly, “Google-like” informatics interface allowing cross-talk between various infrastructures would facilitate discovery of novel cancer biomarkers [13].

Early EHR adoption has been seen in many European nations and domestically in private institutions including Kaiser Permanente and government institutions such as the VA hospital system, and increasingly widespread and rapid adoption in the U.S. continues as a variety of vendors distribute EHR systems to medical practices of all sizes [14]. According to the National Center for Health Statistics data, EHR use by office-based physicians has risen from 42.0% to 78.4% during the past five years [15]. This trend is corroborated by survey data from the American Hospital Association, showing that use of EHR systems in U.S. hospitals rose to 44% in 2012, triple the level in 2010 [16,17]. U.S. Department of Health & Human Services (HHS) data [18] show that health care providers participating in federal EHR Incentive Programs reported increasing use of a wide variety of EHR programs (Table 1), pointing toward an impending complete transition to electronic data. While peer-reviewed data of the myriad of EHR vendors is limited, analysis by private groups demonstrate a fragmented market based on factors such as ease of learning, ease of use, and practice size (Table 1). This growth is emerging despite barriers to physician adoption of EHRs, which include financial (high startup/upkeep costs), technical (computer skill deficits), time (ease of learning the system), and legal (security concerns) barriers to EHR implantation (Table 1) [19].

**Table 1:** **Overview of popular electronic health record vendors with various measures of market share and user satisfaction**

**10 most widely used EHR vendors (according to 2013 % MU attestation)**	**% of providers using EHR vendor as primary in office- based setting in April 2011 [** 18 **]**	**% of providers using EHR vendor as primary in office- based setting in December 2013 [** 18 **]**	**Change in % vendor usage, Dec. 2013 vs. April 2011**	**% of eligible hospitals using EHR vendor as primary in April 2011 [** 18 **]**	**% of eligible hospitals using EHR vendor as primary in December 2013 [** 18 **]**	**Change in % Vendor usage by Hospitals, Dec. 2013 vs. April 2011**	**Ranking for “Ease of documentation of care” out of 31 EHR systems [** 80 **]**	**Ranking for “Ease to learning system” [** 80 **]**	**Ranking on physician satisfaction [** 80 **]**	**Medscape survey 2012, best ranked overall [** 81 **]**	**Medscape survey, % respondents citing as most widely used [** 81 **]**	**% Market share for practices of 1–3 physicians [** 82 **]**	**% Market share for practices of 4–10 physicians [** 82 **]**	**% Market share for practices of 11+ physicians [** 82 **]**
**Epic systems corporation**	12.1%	19.6%	+7.6%	10.2%	15.1%	+4.9%	10 th	18 th	9 th	6 th	22%	6.4%	15.5%	25.6%
**Allscripts**	13.0%	11.0%	−2.0%	3.4%	3.9%	+0.5%	15 th/21 st/23 rd	16 th/24 th/29 th	17 th/24 th/30 th	13 th	10%	11.0%	15.2%	15.3%
**eClinicalWorks LLC**	12.4%	8.6%	−3.8%	N/A	N/A	N/A	11 th	12 th	11 th	8 th	6%	13.8%	11.8%	6.4%
**NextGen healthcare**	9.6%	8.2%	−1.4%	0.6%	1.4%	+0.8	27 th	30 th	26 th	17 th	5%	5.5%	5.8%	5.8%
**GE Healthcare**	6.1%	6.5%	+0.5%	0.3%	0.4%	+0.1	14 th/17 th	19 th/20 th	13 th/21st	10 th	6%	6.2%	11.6%	7.7%
**Cerner corporation**	1.7%	3.7%	+2.1%	15.1%	14.4%	−0.7	N/A	N/A	N/A	11 th	9%	N/A	5.8%	15.6%
**Practice fusion**	1.4%	2.9%	+1.5%	N/A	N/A	N/A	9 th	3 rd	7 th	2 nd	2%	N/A	N/A	N/A
**Vitera healthcare solutions, LLC (Intergy)**	4.9%	2.4%	−2.5%	N/A	N/A	N/A	22 nd	21 st	19 th	N/A	N/A	3.3%	3.6%	N/A
**McKesson**	2.5%	2.1%	−0.4%	7.5%	9.5%	+2.0	N/A	N/A	N/A	16 th	2%	4.1%	N/A	2.4%
**Greenway medical technologies, lnc. (successEHS)**	3.1%	2.2%	−0.9%	0.0%	0.0%	0.0	28 th	28 th	22 nd	N/A	1%	N/A	N/A	N/A

“Provider” is defined as a health care professional who has (1) registered for the CMS Medicare or Medicaid EHR Incentive Programs, (2) attested to Meaningful Use as part of the CMS Medicare EHR Incentive Program, or (3) is receiving technical assistance from the ONC REC Program in order to meet the milestones of the CMS EHR Incentive Programs. “Eligible hospital” is defined as a non-acute care hospital [16]. Primary vendors have EHR products in a provider’s EHR system that meet the majority of “Meaningful Use” criteria; are the sole vendor of EHR products for the provider; or are vendors of a complete EHR product as reported in the Certified EHR Product List.

The rush to adopt EHR has led to vast amounts of patient data entering the digital realm. A critical question concerns how to best utilize this information to improve patient outcomes. A systematic review of the current impact of EHRs on a primary care healthcare system in 2011 showed that EHRs provided “structural and process benefits”, but few data indicated improved patient outcomes [11]. Positive effects found were that EHRs: 1) increased completeness of patient encounters, 2) encouraged patient questions, 3) prevented confusion due to illegible handwriting, and 4) bolstered confidence of clinicians in the EHR system [11]. Negative or adverse effects of EHRs were: 1) no significant difference in total numbers of patient office visits, 2) increased duplicate order entries, 3) increased incidence of insufficient EHR training, and 4) workflow disturbances due to system crashes [11]. Randomized controlled studies have found EHRs may negatively affect physician visualization of patient non-verbal cues [11]. Current understanding of how EHRs truly affect patient care is immature, but improved EHR practices, standardization, and training to correct deficiencies will optimize the effectiveness of EHRs.

## EHRs have laid the foundation for clinical decision support systems

The lack of clear evidence for positive effects of EHR use on clinical outcomes suggests that EHR adoption is only a first step toward improving patient healthcare through informatics. To achieve improved outcomes via EHR use, the best methods to digitize the massive amount of data must be identified. The National Cancer Institute’s now-retired caBIG program and its successor the National Cancer Informatics Program (NCIP) serve as models for integration of large cancer data sets. The development and enhancement of open-source standards has enabled cancer researchers to access up-to-date clinical data, genotypic data, and familial inheritance data for use in designing studies and informing decisions [20]. Clinically, EHRs are providing a wealth of information to researchers and physicians, and early adopters have begun to integrate clinical decision support systems (CDSS), which benefit from the network of information provided by EHRs. Implemented CDSS include reminder boxes for patient follow up, warning systems for deadlines for data submission, and diagnostic suggestions.

## The need for CDSS for precision medicine

**Figure 1:**
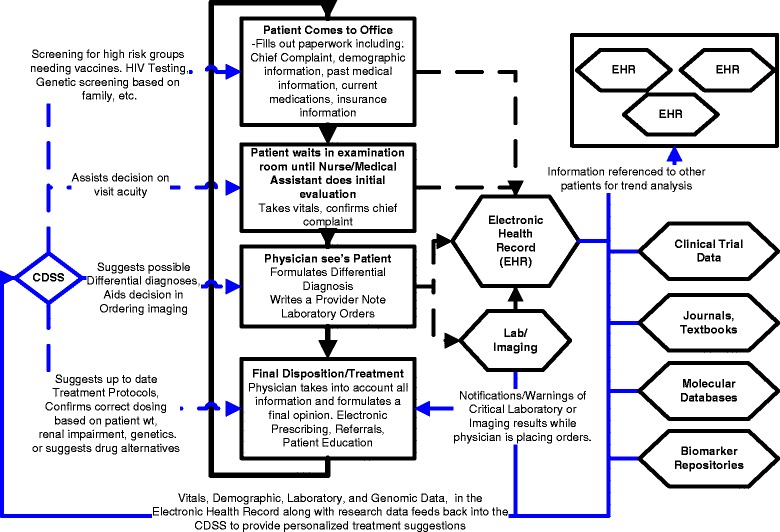
**Typical office-visit workflow, highlighting points at which CDSS may improve care.** Electronic health records act as a reservoir of information used by clinicians and clinical decision support systems to plan healthcare. Along with this, information research data feeds back into the workflow allowing a self-improving cycle of information exchange. CDSS then affects many stages of the office visit and optimizes patient care through warnings, reminders, and suggestions.

CDSS are tools that incorporate established clinical knowledge and updated patient information to enhance patient care; they encompass an array of strategies supporting a variety of topics [21]. CDSS are designed to assist the physician-patient encounter at multiple points from initial consultation to diagnosis to follow up (Figure 1). Expectations are that properly equipped CDSS will significantly benefit patient care at all levels.

CDSS promise to alleviate the ever intensifying time demands upon physicians. Since the Affordable Care Act has been in place, 46% of surveyed emergency medicine physicians have seen increased numbers of patients in emergency rooms [22]. The American Academy of Family Physicians estimates that the number of office visits will increase from 462 million in 2008 to 565 million by 2025 [23]. The American Association of Medical Colleges (AAMC) expects a shortage of 130,600 physicians by 2025 [24]. The Social Security Administration reports that 10,000 baby boomers reach retirement each day [25]. The intensifying divide between supply of physicians and demand on their time highlights the need for properly designed CDSS.

Constraints on time combined with constantly-evolving standards of care contribute to physician errors and delayed clinical decisions [26], which directly and adversely impact medical economics (physician error is the largest contributor to ambulatory malpractice claims costing an average of $300,000 per claim) [27]. Error rates have diminished during the past 40 years, but may still be as high as 24.4% [28]. In a 2009 analysis, 32% of medical errors were due to inadequate time for patient assessment, resulting in less accurate diagnosis, weighing or prioritizing less significant analyses, or not recognizing urgency or complications [26]. Even in a modern clinical setting supported with use of EHRs, 78.9% of diagnostic errors were related to breakdowns in the patient/physician encounter, including 1) failure to order required testing, 2) problems in attaining accurate medical history, 3) inadequate patient examinations by physicians, and 4) inadequate review of available documentation [29]. One study showed missing electronic documentation of differential diagnoses on the index visit in 81.1% of cases [29]. A recent report showed that 74 percent of misdiagnoses involved cognitive errors by the physician. The most common errors were associated with “premature closure”, the tendency to stop considering other possibilities after reaching a diagnosis [30]. Over a 25 year period, diagnostic errors accounted for 28.6% of US malpractice claims [31].

These statistics suggest that current EHRs are populated with data points and information but have not improved understanding or knowledge. Patient data require sequential processing by physicians to reach correct clinical conclusions, and for these conclusions to become knowledge. These issues highlight the need for EHR-based CDSS to translate clinical data points and information into knowledge that can be readily used by time-constrained physicians—knowledge that would decrease the incidence of errors.

CDSS have been implemented in pharmacology, pharmacy, pharmacogenomics, and pathology. Well-characterized CDSS that assess renal function, pregnancy status, duplicate order entry, drug allergy checking, and determining whether a drug choice and dose is unusual in the context of a specific diagnosis are currently used to prevent errors in pharmaceutical dosing, drug-drug interactions, drug-pregnancy interactions, and other medication-related parameters [32]. In a 1994 systematic review of the topic, such drug-prescribing and interaction-warning systems have improved patient outcomes through decreased adverse effects and dosing errors [33]. One computerized order entry support system for renal-toxic and renal-cleared medications increased renal function-appropriate dosing and decreased the median length of stay by half a day [34]. These systems serve a critical need, accounting for constantly-changing pharmaceutical guidelines and interactions and the substantial numbers of subspecialty physicians interacting with an individual patient. One prospective study showed an 86% decrease in serious medication errors after the implantation of CDSS [35]. Case studies have identified increased pathogen susceptibility to selected antimicrobial agents, decreased rates of adverse drug effects, toxic drug levels, and bleeding events on patients taking anticoagulants [33].

CDSS are particularly well-suited to predicting drug-dosing complications associated with drugs that interact with the cytochrome P450 system, which metabolizes drugs that have narrow therapeutic windows (*e.g.,* Warfarin). Future applications of CDSS will optimize drug and dosage selection using low-cost genomic analysis to predict drug metabolism based on individual patient genetic makeup [36]. A CDSS tailored to this purpose will require a large initial data set, but once established will permit safer, evidence-based optimal treatment and dosing for patients.

Pathologists have swiftly adopted CDSS because the pathology report serves as a critical decision point for diverse specialties from preventive medicine to surgery. Advances in histopathological analysis, EHR documentation, and cellular biology techniques have allowed for acquisition of large amounts of digitized patient data and, subsequently, new workflows in the digital pathology laboratory (Table 2) [37]. One such CDSS allows pathologists to pool prostate cancer data and provides prognostic and decision making tools (Table 2) [38]. Another, BRCAPRO, is a software program that predicts probabilities of carrying a deleterious mutation in the breast cancer genes BRCA1 and/or BRCA2 based upon patient cancer status and family history of breast and ovarian carcinoma [39]. BRCAPRO is highly sensitive for screening, and illustrates the immense potential of clinical informatics to guide preventive medicine and improve patient outcomes [39]. These examples in pharmacology and pathology, along with others highlighted in Table 2, illustrate the potential benefits of informatics for clinical decision support.

**Table 2:** **Publications in pathology CDSS implementation**

**CDSS Manuscript title**	**Topic focus**	**Purpose**	**Conclusions of study**
**CMDX©-based single source information system for simplified quality management and clinical research in prostate cancer [** 38 **]**	Prostate Cancer	Created a system used to store topographical information about prostate sample biopsy specimens to create heat-maps for areas of most likely to be prostatic carcinoma, essential for clinical decision-making, prognosis, and research.	Between 2010 and 2011, generated 259 biopsy case reports uploaded to the database with 100% data completeness and a source-to-database error of 10.3 per 10,000 fields. Serves as an implementation of pathology data sharing through a healthcare information system.
**A Teaching Database for Diagnosis of Hematologic Neoplasms Using Immunophenotyping by Flow Cytometry [** 83,84 **]**	Hematologic Neoplasms	Developed of web-enabled relational database, by pooling literature for cell surface marker definitions of 37 hematologic neoplasms. Using this expression profile, an algorithm was created by pathologists to assist in teaching flow cytometry diagnosis of hematologic neoplasms	Algorithm for identifying hematologic marker expression patterns validated using 92 clinical cases with an identification success rate of 89%. Tool has been used by pathologists-in-training to develop flow cytometry interpretation skills.
**Bayesian belief network for the Gleason patterns in prostatic adenocarcinoma: development of a diagnostic decision support system for educational purposes [** 85 **]**	Prostate Cancer	Developed a Bayesian belief network (BBN) for Gleason grading of prostate adenocarcinoma, to allow subjective evaluation of prostatic carcinoma slides by computer.	As histological diagnosis of prostate carcinoma is often produces wide inter-interpreter variability. This tool serves as a decision support tool to interpret descriptive terms in pathology reports and accurately determine tumor grading.
**A computer-based diagnostic and prognostic system forassessing urinary bladder tumour grade and predicting cancer recurrence [** 86 **]**	Bladder Cancer	Designed a decision support system, employed by pathologists in microscopic observation of tissue samples and measurements of nuclear characteristics, allowing automatic assessment of urinary bladder tumor grade and cancer recurrence probability.	The system employed classified tumors with an accuracy of 82%, 80.5%, and 93.1% for tumors of grade I, II, and III. Suggested prognosis in 72.8% of samples with a confidence of 74.5%.
**Comprehensive graphic-based display of clinical pathology laboratory data [** 87 **]**	Information Sharing	Established a graphic-based computerized system for display of clinical pathology data to permit improved data access and sharing.	By displaying laboratory data in a graphical manner, designers aimed to create a user-friendly system meant to highlight pertinent trends necessary for decision management.
**Electronic reminders for pathologists promote recognition of patients at risk for Lynch syndrome: cluster-randomised controlled trial. [** 88 **]**	Colorectal cancer	Deployed a new guideline to improve recognition of patients at risk for Lynch syndrome by a multidisciplinary team of surgeons, pathologists and clinical geneticists.	An electronic reminder system for pathologists increasing identification of patients at high risk for Lynch syndrome by 18% when compared to the control arm.
**Image-guided decision support system for pathology [** 89 **]**	Hematologic Neoplasms	Developed an image based retrieval system containing 261 digitized images of lymphocyte disorders and regular lymphocytes. Queries to the system extract features of the histopathological images for comparison and identification.	Based solely on morphological characteristics, the analysis system performed better than humans in identifying certain lymphoproliferative disorders according to a ten-fold cross-validated confusion matrix.

Selected manuscripts focused upon clinical decision support for pathologists. Ranging from image identification to computerized reminders, the potential for decision support is broad. The purpose of each support system and a summary of conclusions are also noted.

## Why current CDSS are inadequate and the role they must fill

Despite promising initial data from the two aforementioned fields, the majority of CDSS have not provided features beyond general alerts, reminders, summary dashboards, and automated information retrieval systems [40,41]. A majority of United States hospitals have yet to implement any form of CDSS. “Comprehensive” EHR systems are those that include decision support features (clinical guidelines, clinical reminders, drug-allergy alerts, drug-drug interaction alerts, drug laboratory interaction alerts, drug-dosing support); only 1.5% of 2952 hospitals surveyed achieved a “comprehensive” classification [42]. Few studies of CDSS have shown any improvement in outcomes, and any such effects seen have achieved only low statistical significance [40]. A meta-analysis of 148 randomized clinical trials on CDSS implementation found that only 20% influenced clinical outcomes [41]. Of these, improvements were seen in morbidity outcomes such as number of hospitalizations, surgical site infections, cardiovascular events, and deep vein thrombosis, but there was little effect on mortality or pharmacologic adverse events [41], suggesting that CDSS will need improvement before they can routinely provide clinically meaningful knowledge.

Scientific and clinical data sets are typically located in extremely large files located in different databases worldwide (“information silos”). To convert endless data points and relevant information into forms easily reviewed by physicians and researchers, information silos must be connected. Intelligent algorithms must apply standardized languages/phenotypes to decide which pieces of information may be relevant to a specific query. If the vast amount of patient data currently present in hospital EHR databases is considered as another information silo, then how would connections be made from patient data to primary literature for both scientific and clinical use? A truly useful CDSS would combine data points and information generated from thousands of hours of research, clinical assays, blood work, and follow up data to reach the clinical endpoint. Bridging of knowledge between these realms embodies the idea of collaboration in medicine.

## Evolution from data, to processed information, to integrated knowledge, to expert systems

In the early days of computing, digitized data were stored in files, later in databases, and now in data warehouses and, via the internet, in “the cloud”. Data capacity and storage have become massive, paving the way for current “big data” paradigms and initiatives that promise to provide answers to increasingly complex and challenging scientific and medical questions. The key barriers to progress are the disparate and dispersed nature of “big data”. The challenge is to develop means for integrating or querying across the relevant information silos to gain needed insights and problem solutions.

Scientific research in diverse arenas yields in-depth results representing new data and interpretive information that is generally distributed to other scientists via publications in scientific journals and entry into databases. The vertically-organized nature of such knowledge tends to sustain data within silos that contribute to access barriers, resisting facile integration and querying of data. Therefore, to effectively leverage new scientific discoveries, and broaden understanding by scientists working in disparate fields, a tool to interrogate data across the existing information silos is critically needed.

A common and useful method for integrating disparate data sources is to apply standard vocabularies and data formats, with the goal of reducing complexity and technical barriers to their integration across fields of specialization. Fortunately, scientific information is extremely standardized, and data formatting is also guided and regulated by US national agencies, including the National Institute of Standards and Technologies (NIST) and the National Institutes of Health (NIH) [43]. Standards for clinical data points and information contained in EHRs and various clinical reports in forms of paper-based physician or patient charts have been developed by Health Level Seven International (HL7), the World Health Organization International Classification of Disease (ICD9, ICD10, and ICD-O), and the International Health Terminology Standards Development Organization (SNOMED) [44]. To permit integration of genomic data into CDSS and to describe data around biological concepts, integrating resources such as Gene Ontology (GO) and HUGO Gene Nomenclature will be necessary.

As knowledge advances, the terms used to communicate such knowledge must be aligned and integrated into the knowledge foundation represented by accepted standards. Also, new standards must be applied to existing data in a manner allowing comparisons between current and historical data. Ongoing discussions required to develop such standards are significant and substantive, and can be tedious, arduous, and controversial. Continuously evolving standards and data are challenges to large-scale adoption, but effective implementation of these ‘internationally accepted’ standards is required to accelerate the use of CDSS and to enhance the quality of patient care.

New research platforms such as those driven by various genomic and other “-omic” technologies generate massive amounts of raw data that require analysis using biostatistical methods and a range of visualization tools. Information from new platforms is often difficult to relate to data generated by other platforms. To address this issue, commercial software companies have developed suites of powerful tools focused on platform-specific data (Table 3). For example, large datasets of gene expression experimental results are stored in the NCBI Gene Expression Omnibus (GEO) [45]. Several organizations and companies have developed effective and innovative ways to mine disparate gene expression data in GEO, which enables researchers to access and analyze valuable information (Table 3). Such domain-specific systems are built on database technologies that allow information from different databases to be logically linked, providing additional and enriched capabilities to researchers.

**Table 3:** **Overview of software systems designed to integrate data from multiple databases**

**Database integration system**	**Purpose**	**License**	**Update method**	**# of databases**	**Databases integrated**	**Company**
**Atlas [** 90 **]**	“A biological data warehouse called Atlas that locally stores and integrates biological sequences, molecular interactions, homology information, functional annotations of genes, and biological ontologies”.	Open Source	Manual	13	GenBank, RefSeq, UniProt, Human Protein Reference Database (HPRD), Biomolecular Interaction Network Database (BIND), Database of Interacting Proteins (DIP), Molecular Interactions Database (MINT), IntAct, NCBI Taxonomy, Gene Ontology (GO), Online Mendelian Inheritance in Man (OMIM), LocusLink, Entrez Gene and HomoloGene	British Columbia University - Vancouver, BC
**Biowarehouse [** 91 **]**	“An open source toolkit for constructing bioinformatics database warehouses using the MySQL and Oracle relational database managers. Integrates multiple public bioinformatics databases into a single relational database system within a common bioinformatics schema”.	Open Source	Dependent on the individual databases	12	ENZYME, KEGG, BioPax, Eco2dbase, Metacyc, Mage-ML and BioCyc, UniProt, GenBank, NCBI Taxonomy, CMR databases, and Gene Ontology.	Stanford Research Institute – Menlo Park, Ca
**Columba [** 92 **]**	“Facilitates the creation of protein structure data sets for many structure-based studies. It allows combining queries on a number of structure-related databases not covered by other projects at present”.	Free-Use	Dependent on the individual databases	12	PDB, SCOP, CATH, DSSP, ENZYME, Boehringer, KEGG, Swiss-Prot, GO, GOA, Taxonomy, PISCES	Humboldt-Universität zu Berlin – Berlin Germany
**Systomonas [** 93 **]**	“To provide an integrated bioinformatics platform for a systems biology approach to the biology of pseudomonads in infection and biotechnology”.	Free-Use	Unknown	4	KEGG, Pseudomonas Genome Database v2, PRODORIC, and BRENDA	Technische Universität Braunschweig - Braunschweig, Germany
**Oncomine [** 94 **]**	“A cancer microarray database and web-based data-mining platform aimed at facilitating discovery from genome-wide expression analyses”.	Free-Use, Subscription-based for expanded functionality	Annually for Free Version, Regular data updates for subscription	-	65 Gene expression datasets, from 4700 microarray experiments.	Life Technologies Corporation
**Biomart [** 95 **]**	“BioMart enables scientists to perform advanced querying of biological data sources through a single web interface. The power of the system comes from integrated querying of data sources regardless of their geographical locations”.	Open Source	Unknown	25 (as of 2009), 46 as of 5/2014	Ensembl Genes, Ensembl Homology, Ensembl Variation, Ensembl Genomic Features, Vega, HTGT, Gramene, Reactome, Wormbase, Dictybase, RGD, PRIDE, EURATMart, MSD, Uniprot, Pancreatic Expression Database, PepSeeker, ArrayExpress, GermOnLine, DroSpeGe, HapMap, VectorBase, Paramecium, Eurexpress, Europhenome	Collaboration between many institutes and Universities.
**Ondex [** 96 **]**	“The Ondex data integration platform enables data from diverse biological datasets to be linked, integrated and visualised through graph analysis techniques. Ondex can be used in a number of important application areas such as transcription analysis, protein interaction analysis, data mining and text mining”.	Open Source	Unknown	28	AraCyc, AtRegNet, BioCyc, BioGRID, Brenda, Cytoscape, EcoCyc, GOA, Gramene, Grassius, KEGG, Medline, MetaCyc, O-GlycBase, OMIM, PDB, Pfam, Prolog (limited functionality), SGD, TAIR, TIGR, Transfac, transpath, UniProt, WordNet, ChEBI, ChEMBL, GFF3	Rothamsted Research Harpenden, UK
**InterMine [** 97 **]**	“InterMine is an open-source data warehouse system that facilitates the building of databases with complex data integration requirements and a need for a fast customizable query facility. Using InterMine, large biological databases can be created from a range of heterogeneous data sources, and the extensible data model allows for easy integration of new data types”.	Open Source	Unknown	23	GO Annotation, GO OBO, Treefam, Homologene, OrthoDB, Panther, Ensembl, Compara, BioGRID, IntAct, PSI-MI Ontology, KEGG, Reactome, UniProt, Protein Data Bank, InterPro, PubMed, Ensembl SNP, Chado, Ensembl Core, FASTA, GFF3, OMIM, Uberon	University of Cambridge - Cambridge, United Kingdom
**Scan-MarK [** 65 **]**	“An integrated, growing biomarker repository of over 2,000 breast, ovarian, colorectal, non-Hodgkin’s lymphoma and melanoma biomarkers mined and manually curated by PhD. scientist from full-text papers. Annotations include 33 critical data elements (CDEs) organized in computable Sophic Cancer Biomarker Objects (SCBOs). SCan-MarK allows researchers to mine, explore and expose complex biomarker, disease, treatment, outcome relationships graphically displayed as knowledge networks”.	Free Trial	Manual	30	Examples: TCGA, dbSNP, Cancer Gene Index, Drugbank, PDB, Sophic’s non-redundant Sanger COSMIC, Medline, ENSEMBL, ENZYME, Go, Interpro, Pfam, Pubchem, Unigene, Taxonomy, Uniprot, Refseq, Entrezgene, Reactome Pathway	Sophic Alliance

Displayed are current software solutions whose primary goal is to facilitate research workflow through data-mining algorithms. These software solutions range from open-source to paid subscription, and target specific subgroups of scientists. A common underlying goal amongst these examples is centralization of multiple databases through the use of algorithms and standardization.

The concept of an integrated knowledge environment is based on connecting structured but disparate database repositories with information stored in unstructured, text-based repositories and databases. Narratives and descriptions of specific domains (*e.g*., gene expression profiles, cell signaling pathways, Next Generation Sequencing (NGS), mutations) provide accurate, efficient means for connecting information stored in classic database schemas. In the federated database approach, a metadata layer links disparate databases permitting integrating, mining, and searching all types of information. The metadata is structured information that describes and links the underlying data sources; it describes the data elements, can define the structure of the data, and can define the administrative information such as when the data was generated and by whom. The metadata layer may utilize existing standards or may employ de novo standards appropriate to the underlying data sources [46]. An important metadata concept for scientific and medical data is the ability to assign confidence to the data sources, allowing a user to determine whether the accessed data is actionable in a clinical or research setting. Since data definitions and structures evolve over time, generation of a metadata layer requires a flexible data model that describes the objects and data elements contained in various sources [46,47]. Application of common standards allows disparate information to be mapped so as to expose valid semantic and scientific relationships. Such lateral, cross-cut information views allow relevant relationships to be visualized within network maps. Visualization by network maps provides layers of information about biological activities, disease progression, mechanisms of action, and pathways that can enhance analysis of the evidence necessary to provide input into a CDSS (Figure 1) [48].

## Supercomputers, artificial intelligence, and medicine

IBM’s Watson supercomputer captured the imagination of the world when it beat two reigning champions on the television quiz-show *Jeopardy* in 2011 [49]. This achievement represented a significant milestone in computer science, because the *Jeopardy* game requires abstraction and ‘understanding’ natural language. Due to the structure of the questions, Watson could not simply reference databases of pre-computed questions and answers but had to use the Deep QA architecture, creating hypotheses based on sources, referencing sources for evidence, scoring the evidence, and synthesizing the data into a final ranking [50,51]. The system’s critical function is its analytic process. The computer must weigh relevant parameters for each item of reference material and construct a summarized interpretation yielding a conclusion. For example, how should a particular statement in a diabetes textbook be weighted relative to a recently-published article on diabetes? How should the system resolve conflicting information? One method to address these concerns is to train Watson through experience. Experiential training allows artificial intelligence systems such as Watson to 1) translate data points (first tier) to information (second tier), 2) convert information to clinically relevant and meaningful knowledge (third tier), and to 3) achieve expert understanding (fourth tier).

The current approach is to enter scenarios and case reports that have been annotated by medical professionals highlighting best answers or most likely diagnoses, training the system to recognize classical presentations of disease [52]. This approach is working; in statements by David Ferrucci, Watson principle investigator, Watson is currently out-diagnosing medical residents in selected criteria [53]. Adding training and additional content to Watson has improved its accuracy in answering American College of Physician clinical question sets by 28% [51].

A second approach to training the supercomputer is to feed the system thousands of medical records and diagnoses, enabling it to analyze trends and associations hidden in physician notes, laboratory data, genetic data, and other records. IBM has partnered with speech-recognition software companies, health benefits companies, and other institutions to develop training for CDSS that fully encompasses the entire physician-patient interaction from healthcare outcomes to EHRs [54-56]. News releases by IBM state this approach is being used in the development of personalized cancer therapy at MD Anderson [55]. Nonetheless, system training incorporating only clinical presentations and phenotypes in the absence of scientific and biomarker data will provide a less than desirable outcome. The ultimate goal will be to incorporate both clinical and scientific data, which will facilitate analysis that fully encompasses the entire laboratory-physician-patient interaction from genotype to clinical outcome.

## Why is Watson important in clinical medicine?

Watson’s implementation marks the development of the first large-scale, integrated CDSS capable of processing both structured and unstructured information to suggest possible differential diagnoses. This artificial intelligence system addresses two primary reasons for physician error: 1) premature closing of diagnosis (defined earlier) and 2) failure to consider all other possibilities [31]. While peer-reviewed literature is not available due to the youth of this partnership, Wellpoint Inc. states Watson’s support of utilization management decisions in 1,500 cases has shown encouraging results [56].

As computing technology moves forward, the unprecedented algorithm enabling interpretation of natural language by CDSS systems will directly assist analysis of primary literature, reducing the need for manually-curated sets of rules that are often limited in topic or scope. By allowing systems such as Watson to analyze information such as patient notes, laboratory data, genotype data, and familial inheritance data, individualized clinical and molecular profiles of each patient can be assembled. The individualized patient profile can then be compared to the thousands of other patient EHRs to identify similarities and associations, thus, elucidating trends in disease course and management. Each new and validated association extends the foundation of the Watson architecture and facilitates increased confidence in subsequent output for diagnoses. These strengths and progress represent a significant advance in the field of clinical informatics.

Although it has received widespread recognition and media coverage, IBM’s Watson is not alone; a number of other players in the field of medical informatics supercomputing have also emerged. Alternative analytic systems such as Hadoop, an open source software framework for large dataset analysis, are already being used in BLAST multiple sequence alignment and in the topological analysis of immense genotype datasets [57]. The advance in semantic-oriented analytic software is also exemplified in the development of Wolfram Alpha, a knowledge engine infrastructure used to evaluate and interpret datasets ranging from higher mathematics to engineering [58]. Interpretation of English language queries by Wolfram Alpha draws parallels to the natural language processing capabilities of IBM’s Watson. Application to the field of clinical informatics seems inevitable; asking Wolfram Alpha “How should I treat the common cold?” returns data on the most commonly prescribed drugs, sorted by drug class. In fact, Wolfram’s program language already includes functions to manipulate genome data and parse protein databases [59]. The undeveloped nature of this field has opened the door to the emergence of a wide variety of supercomputing algorithms and frameworks.

While promising, this technology is still new, and continuing input from clinicians, patients, and other stakeholders will be essential for correctly implementing the technology [60]. System development is costly, and requires substantial hands-on training for optimal use. According to Wellpoint Inc., nurses have already spent over 14,700 hours training Watson [61]. A critical question is whether such costs are justified. Does Watson or similar artificial intelligence-based CDSS effectively and efficiently improve patient outcomes?

The prospect of artificial intelligence-based CDSS and their role as the umbrella of all scientific and clinical data depends on a dedicated roadmap and investments from the government and public and private sectors. Deployment of a mature Level 4 EHR is expected to cost $28 billion dollars per year over a 10 year adoption period [62]. Private research firms estimate Watson’s initial development costs to be somewhere between $900 million and $1.8 billion dollars [63]. Significant investments may be required to achieve widespread use of artificial intelligence-based CDSS in the clinical setting.

## How data integration affects medical research

Improved patient outcomes are the most immediate endpoint of CDSS, but information and data output need to flow back to researchers. As in clinical medicine, time and manpower in laboratories are precious resources. Integration of the biomedical community through translational bioinformatics is important [12,64]. Systems such as Hugenet, an aggregation of genomic data with environmental and public health registries [64], and SCanMarK Explorer, an integrated knowledge environment that includes a manually curated cancer biomarker database [65], have been created within focused specialized topic areas. These and other similar software platforms provide researchers with valuable and integrated databases upon which models can be designed and research furthered (Table 3) [12]. Despite these advances, a pressing concern is the task of keeping these integrated databases up-to-date. Astonishingly, the current state-of-the-art for updating many of these integrated knowledge environments is nothing more than manual data entry (Table 3).

Major cancer centers at Massachusetts General Hospital and Memorial Sloan Kettering are already using next generation sequencing technologies to integrate patient specific genomic information with research data to generate the foundation of personalized cancer treatment plans [66]. Use of EHRs will have become mandatory in U.S. hospitals in 2015. The vast amount of information that becomes available will need to be integrated. As more outcomes data become available, researchers will need access to it [64]. This bidirectional flow of information promises to decrease the time between laboratory discovery and clinical implementation. Electronic integration between clinics and scientists can expedite research impacts. For example, Vioxx (Rofecoxib) was approved by the FDA in 1999 and released by Merck for pain management, with the promise of decreased adverse gastrointestinal symptoms. However, in 2003, an increased risk of myocardial infarctions within 90 days of beginning treatment was identified, ultimately causing worldwide withdrawal of the drug in 2004 [67]. The discovery and quick action taken was primarily due to Kaiser Permanente’s early support of patient databases and registries, which allowed researchers and physicians to assess long-term patient outcomes. The system allowed physicians to immediately access data for research and to receive instant feedback [14].

## Concerns in digitalization and integration of health records and databases

The rapid expansion of computerization into the healthcare arena raises many ethical and legal concerns with respect to privacy, confidentiality, and data exchange. From a moral perspective, the most pressing concern to the general public and healthcare systems alike is whether genotype data and more generally digitized data can be made truly secure. The problem with this is that genotype data is inherently not private [68]. The identity of a patient with a rare genetic condition would easily be identified amongst a database of persons without said disease. This engenders concerns regarding potential controversial practices such as discrimination based on genetics against patients by insurance companies [68,69]. These issues have been recognized by the US Government, and legislation such as the Genetic Information Nondiscrimination act of 2008 and the 2010 Patient Protection and Affordable care act include provisions to protect against genetic discrimination [70]. Even the National Institutes of Health has removed SNP data from publically available databases in an effort to protect research participant privacy [71]. These actions are a promising start, but the ethical ramifications of merging databases containing genomic information with databases containing patient data have yet to be addressed. How should researchers handle informed consent in the case of incidental findings on patient genomic data [72]? How should genomic data shared between researchers be handled? The issue of confidentiality must be properly addressed in order to allow continued genomic research and collaboration [69].

From a technological standpoint, a survey of US Healthcare institutions by a private data security group showed that up to 40% of those institutions lacked confidence in the security of health information exchange systems including EHRs [73]. Understanding how to ensure privacy and security is a key challenge. The experience has been rocky. A data breach in 2010 exposed data of 6,800 patients to internet search engines [74]. A breach in 2013 exposed data of 32,000 patients due to failure of a security firewall [75]. Complicating matters is the wide range of mobile devices that can store patient data. A stolen physician’s laptop in 2012 contained patient data [76]. Each of these breaches resulted in costly settlements. Companies have stepped forward to address the security needs of EHRs. For example, BlackPhone, a mobile smartphone focused on security and data exchange [77], decreases electronic exposure risk through encrypted voice calls and texts. Such technologies have natural applications to the sensitive data routinely transferred within hospitals, and their implementation may help prevent costly data breaches and HIPAA violations.

Finally, from a logistical perspective, the wide variety of implementations and permutations of electronic health records will challenge data exchange both amongst healthcare providers and between healthcare providers and researchers. The benefits of healthcare information exchange are clear. Reducing: laboratory redundancy, imaging redundancy, and administrative costs are a few of the theoretical advantages [62]. As a result, patient safety stands to improve in a number of different ways [78]. The problem lies in that electronic health systems were not built with the goal of being interoperable, they were built with the goal of meeting meaningful use standards [79]. Furthermore, data on the exact structure and syntax by which these different EHR systems operate is limited [4]. Due to these differences, a fundamental framework for connecting these silos of healthcare information is lacking, and therefore, poses one more challenge to the goal of an integrated information network.

## Conclusions

We have entered an era where use of computers, tablets, and smart phones allow the capture of nearly all information in digital format. The challenge is not “How can we collect all of these data?” but “How can we integrate, secure, and utilize this large amount of data via CDSS to improve clinical outcomes?” Computer-based systems that can host all scientific and clinical data are critical to generating the end-point knowledge that is directly relevant to improved understanding and management of disease for each patient. As supercomputing, science, and medicine continue to converge, the conversion of data to knowledge, and sharing of such knowledge, has become a crucial component for achieving precision medicine and personalized patient care.

## Abbreviations

CDSS: Clinical decision support system
EHR: Electronic health record
HITECH: Health information technology for economic and clinical health act
HIPAA: Health insurance portability and accountability act
FDA: Food and drug administration
CMS: Centers for medicare and medicaid services
MU: Meaningful use

## References

[1] “Clinical Informatics”. Home. N.p., n.d. Web. 29 Dec. 2013. <http://www.amia.org/applications-informatics/clinical-informatics>.

[2] “New Clinical Informatics Subspecialty and First Class of Diplomates Signal Pivot in Healthcare Delivery”. Home. N.p., n.d. Web. 29 Dec. 2013. <http://www.amia.org/news-and-publications/press-release/new-clinical-informatics-subspecialty-and-first-class-diplomates>.

[3] “Translational Bioinformatics”. Home. N.p., n.d. Web. 29 Dec. 2013. <http://www.amia.org/applications-informatics/translational-bioinformatics>.

[4] Häyrinen K, Saranto K, Nykänen P (2008). Definition, structure, content, use and impacts of electronic health records: a review of the research literature. Int J Med Inform.

[5] Katayama T, Arakawa K, Nakao M, Ono K, Aoki-Kinoshita KF, Yamamoto Y (2010). The DBCLS BioHackathon: standardization and interoperability for bioinformatics web services and workflows. The DBCLS BioHackathon Consortium*. J Biomed Semant.

[6] Defrancesco L (2012). Life Technologies promises $1,000 genome. Nat Biotechnol.

[7] Steinbrook R (2009). Health care and the American recovery and reinvestment act. N Engl J Med.

[8] Friedman CP, Wong AK, Blumenthal D (2010). Achieving a nationwide learning health system. Sci Transl Med.

[9] Blumenthal D (2010). Launching HITECH. N Engl J Med.

[10] Ash JS, Bates DW (2005). Factors and forces affecting EHR system adoption: report of a 2004 ACMI discussion. J Am Med Inform Assoc.

[11] Holroyd-Leduc JM, Lorenzetti D, Straus SE, Sykes L, Quan H (2011). The impact of the electronic medical record on structure, process, and outcomes within primary care: a systematic review of the evidence. J Am Med Inform Assoc.

[12] Blake PM, Decker DA, Glennon TM, Liang YM, Losko S, Navin N (2011). Toward an integrated knowledge environment to support modern oncology. Cancer J.

[13] Suh KS, Sarojini S, Youssif M, Nalley K, Milinovikj N, Elloumi F (2013). Tissue banking, bioinformatics, and electronic medical records: the front end requirements for personalized medicine. J Oncol.

[14] The Kaiser permanente health record: transforming and streamlining modalities of care. Health Aff. 2009; 28(2): 323–333 10.1377/hlthaff.28.2.323 19275987

[15] Hsiao C-J, Hing E (2014). Use and characteristics of electronic health record systems among office-based physician practices: United States, 2001–2013. NCHS data brief, no 143.

[16] Desroches CM, Charles D, Furukawa MF, Joshi MS, Kralovec P, Mostashari F (2013). Adoption of electronic health records grows rapidly, but fewer than half of US hospitals had at least a basic system in 2012. Health Aff (Millwood).

[17] Charles D, King J, Patel V, Furukawa MF (2013). “Adoption of Electronic Health Record Systems among U.S. Non-federal Acute Care Hospitals: 2008–2012”, ONC Data Brief, no 9.

[18] Department of Health & Human Services (2014). Electronic Health Record Vendors Reported by Health Care Providers Participating in Federal EHR Incentive Programs.

[19] Boonstra A, Broekhuis M (2010). Barriers to the acceptance of electronic medical records by physicians from systematic review to taxonomy and interventions. BMC Health Serv Res.

[20] Von Eschenbach AC, Buetow K (2006). Cancer informatics vision: caBIG. Cancer Inform.

[21] Osheroff JA, Teich JM, Middleton B, Steen EB, Wright A, Detmer DE (2007). A roadmap for national action on clinical decision support. J Am Med Inform Assoc.

[22] 2014 ACEP Polling Survey Results. April 2014. Accessed November 3, 2014.

[23] Petterson SM, Liaw WR, Phillips RL, Rabin DL, Meyers DS, Bazemore AW (2012). Projecting US primary care physician workforce needs: 2010–2025. Ann Fam Med.

[24] Association of American Medical Colleges. GME Funding: How to Fix the Doctor Shortage. Available at: https://www.aamc.org/advocacy/campaigns_and_coalitions/fixdocshortage/.

[25] Social Security Administration Annual Performance Plan for fiscal year 2012. Available at: http://www.ssa.gov/agency/performance/2012/APP%202012%20508%20PDF.pdf Accessed November 3, 2014.

[26] Schiff GD, Hasan O, Kim S, Abrams R, Cosby K, Lambert BL (2009). Diagnostic error in medicine: analysis of 583 physician-reported errors. Arch Intern Med.

[27] Singh H, Graber M (2010). Reducing diagnostic error through medical home-based primary care reform. JAMA.

[28] Shojania KG, Burton EC, Mcdonald KM, Goldman L (2003). Changes in rates of autopsy-detected diagnostic errors over time: a systematic review. JAMA.

[29] Singh H, Giardina TD, Meyer AN, Forjuoh SN, Reis MD, Thomas EJ (2013). Types and origins of diagnostic errors in primary care settings. JAMA Int Med.

[30] Graber ML, Franklin N, Gordon R (2005). Diagnostic error in internal medicine. Arch Intern Med.

[31] Sabertehrani AS, Lee H, Mathews SC, Shore A, Makary MA, Pronovost PJ (2013). 25-Year summary of US malpractice claims for diagnostic errors 1986–2010: an analysis from the national practitioner data bank. BMJ Qual Saf.

[32] Kuperman GJ, Bobb A, Payne TH, Avery AJ, Gandhi TK, Burns G (2007). Medication-related clinical decision support in computerized provider order entry systems: a review. J Am Med Inform Assoc.

[33] Kaushal R, Shojania KG, Bates DW (2003). Effects of computerized physician order entry and clinical decision support systems on medication safety: a systematic review. Arch Intern Med.

[34] Chertow GM, Lee J, Kuperman GJ, Burdick E, Horsky J, Seger DL (2001). Guided medication dosing for inpatients with renal insufficiency. JAMA.

[35] Bates DW, Teich JM, Lee J, Seger D, Kuperman GJ, MaLuf N (1999). The impact of computerized physician order entry on medication error prevention. J Am Med Inform Assoc.

[36] Welch BM, Kawamoto K (2013). Clinical decision support for genetically guided personalized medicine: a systematic review. J Am Med Inform Assoc.

[37] Stathonikos N, Veta M, Huisman A, Van Diest PJ (2013). Going fully digital: perspective of a Dutch academic pathology lab. J Pathol Inf.

[38] Eminaga O, Abbas M, Hinkelammert R, Bettendorf O, Eltze E, Ozgur E (2012). CMDX©-based single source information system for simplified quality management and clinical research in prostate cancer. BMC Med Inform Decis Mak.

[39] Berry DA, Iversen ES, Gudbjartsson DF, Hiller EH, Garber JE, Peshkin BN (2002). BRCAPRO validation, sensitivity of genetic testing of BRCA1/BRCA2, and prevalence of other breast cancer susceptibility genes. J Clin Oncol.

[40] Jaspers MW, Smeulers M, Vermeulen H, Peute LW (2011). Effects of clinical decision-support systems on practitioner performance and patient outcomes: a synthesis of high-quality systematic review findings. J Am Med Inform Assoc.

[41] Bright TJ, Wong A, Dhurjati R, Bristow E, Bastian L, Coeytaux RR (2012). Effect of clinical decision-support systems: a systematic review. Ann Intern Med.

[42] Jha AK, Desroches CM, Campbell EG, Donelan K, Rao SR, Ferris TG (2009). Use of electronic health records in U.S. hospitals. N Engl J Med.

[43] National Institute of Standards and Technology. Available at: http://www.nist.gov/. Accessed November 3, 2014.

[44] Averill RF, Mullin RL, Steinbeck BA, Goldfield NI, Grant TM (1998). Development of the ICD-10 procedure coding system (ICD-10-PCS). J AHIMA.

[45] Edgar R, Domrachev M, Lash AE (2002). Gene expression omnibus: NCBI gene expression and hybridization array data repository. Nucleic Acids Res.

[46] National Information Standards Organization. Understanding Metadata. 2004; NISO Press. URL:http://www.niso.org/standards/resources/UnderstandingMetadata.pdf.

[47] Sen A (2004). Metadata management: past, present and future. Decis Support Syst.

[48] Barabási AL, Gulbahce N, Loscalzo J (2011). Network medicine: a network-based approach to human disease. Nat Rev Genet.

[49] Jeopardy! Episodes 27.111–3; National Broadcasting Company. 2011.

[50] Ferrucci D, Brown E, Chu-Carroll J, Fan J, Gondek D, Kalyanpur AA (2010). Building watson: an overview of the deep QA project. AI Mag.

[51] Ferrucci D, Levas A, Bagchi S, Gondek D, Mueller ET (2013). Watson: beyond jeopardy!. Artif Intell.

[52] Weiss SM, Kulikowski CA, Amarel S, Safir A (1978). A model based method for computer-aided decision-making. Artif Intell.

[53] TED. Final Jeopardy! and the future of Watson. http://www.wired.co.uk/news/archive/2013-02/11/ibm-watson-medical-doctor (Accessed April 2012).

[54] IBM to Collaborate with Nuance to Apply IBM’s “Watson” Analytics Technology to Healthcare. IBM News Release 2011. https://blog.blackphone.ch/2015/03/02/mobileworldcongress/.

[55] MD Anderson Taps IBM Watson to Power “Moon Shots” Mission Aimed at Ending Cancer, Starting with Leukemia. IBM News Release. 2013. http://www-03.ibm.com/press/us/en/pressrelease/42214.wss.

[56] WellPoint and IBM Announce Agreement to Put Watson to Work in Health Care. IBM News Release 2011. http://www-03.ibm.com/press/us/en/pressrelease/35402.wss.

[57] Taylor RC (2010). An overview of the Hadoop/MapReduce/HBase framework and its current applications in bioinformatics. BMC Bioinforma.

[58] Weisstein E (2014). Computable data, mathematics, and libraries in mathematica and wolfram alpha. Intell Comput Math.

[59] Wolfram Language and System Documentation Center. Available at: http://reference.wolfram.com/language/. Accessed February 8, 2015.

[60] Arnaout R (2012). Elementary, my dear doctor watson. Clin Chem.

[61] IBM Watson Hard At Work: New Breakthroughs Transform Quality Care for Patients. IBM News Release. 2013. https://www-03.ibm.com/press/us/en/pressrelease/40335.wss.

[62] Walker J, Pan E, Johnston D, Adler-milstein J, Bates DW, Middleton B (2005). The value of health care information exchange and interoperability. Health Aff (Millwood).

[63] 5 billion dollar tech gambles. Available at: http://money.cnn.com/2011/02/14/technology/ibm_jeopardy_watson/. Accessed November 3, 2014.

[64] Sarkar IN, Butte AJ, Lussier YA, Tarczy-Hornoch P, Ohno-Machado L (2011). Translational bioinformatics: linking knowledge across biological and clinical realms. J Am Med Inform Assoc.

[65] Sophic Alliance. A White Paper on Scan-MaRK. http://www.sophicalliance.com/SCan-MarK.php.

[66] Hayden EC (2009). Personalized cancer therapy gets closer. Nature.

[67] Psaty BM, Furberg CD (2005). COX-2 inhibitors–lessons in drug safety. N Engl J Med.

[68] Heeney C, Hawkins N, De Vries J, Boddington P, Kaye J (2011). Assessing the privacy risks of data sharing in genomics. Public Health Genomics.

[69] Greenbaum D, Sboner A, Mu XJ, Gerstein M (2011). Genomics and privacy: implications of the new reality of closed data for the field. PLoS Comput Biol.

[70] Hudson KL (2011). Genomics, health care, and society. N Engl J Med.

[71] Kaye J, Heeney C, Hawkins N, De Vries J, Boddington P (2009). Data sharing in genomics–re-shaping scientific practice. Nat Rev Genet.

[72] Mcguire AL, Joffe S, Koenig BA, Biesecker BB, McCullough LB, Blumenthal-Barby JS (2013). Point-counterpoint. Ethics and genomic incidental findings. Science.

[73] Ponemon Institute. Fourth Annual Benchmark Study on Patient Privacy and Data Security. http://www2.idexpertscorp.com/ponemon-report-on-patient-privacy-data-security-incidents/.

[74] Tremaine DW, Blanchette KR, Greene AH and Williams RL. $4.8 Million – largest HIPAA settlement to date. May 2014. http://www.lexology.com/library/detail.aspx?g=d16341e1-05e5-4df0-820c-4687323a75dc.

[75] Ouellette P. Cogent Healthcare contractor M2ComSys breaches patient data. August 2013. http://healthitsecurity.com/2013/08/12/cogent-healthcare-contractor-m2comsys-breaches-patient-data/.

[76] Freemire J, Wieland JB. $1.5 Million OCR HIPAA Settlement Provides Notice of Increased Enforcement Focus on Mobile Device Security and Encryption. Bloomberg BNA Health IT Law & Industry Report. September 28, 2012.

[77] New Smartphone To Put Privacy And Control First. Press Release. February 10, 2014. https://www.blackphone.ch/new-smartphone-to-put-privacy-and-control-first/.

[78] Kaelber DC, Bates DW (2007). Health information exchange and patient safety. J Biomed Inform.

[79] Bisbal J, Berry D (2011). An analysis framework for electronic health record systems. Interoperation and collaboration in shared healthcare. Methods Inf Med.

[80] Edsall RL, Adler KG (2012). The 2012 EHR user satisfaction survey: responses from 3,088 family physicians. Fam Pract Manag.

[81] Kane L (2012). EHR Report 2012: Physicians Rank Top EHRs. Medscape.

[82] Brookstone AJ, Underwood WS, Barr MS. Market Share and Top 10 Rated Ambulatory EHR products by practice size. AmericanEHR Partners, LLC. July 2011

[83] Nguyen AN, De J, Nguyen J, Padula A, Qu Z (2008). A teaching database for diagnosis of hematologic neoplasms using immunophenotyping by flow cytometry. Arch Pathol Lab Med.

[84] Nguyen A, Wu S, Jalali M, Uthman M, Johnson K, Banez E (2001). A web-based database for diagnosis of haematologic neoplasms using immunophenotyping by flow cytometry. Med Inform Internet Med.

[85] Montironi R, Ball RY, Griffiths DF, Grigor K, Harnden PM, Jarmulowicz M (2008). Bayesian belief network for the Gleason patterns in prostatic adenocarcinoma: development of a diagnostic decision support system for educational purposes. Anal Quant Cytol Histol.

[86] Spyridonos P, Cavouras D, Ravazoula P, Nikiforidis G (2002). A computer-based diagnostic and prognostic system for assessing urinary bladder tumour grade and predicting cancer recurrence. Med Inform Internet Med.

[87] Henry JB, Kelly KC (2003). Comprehensive graphic-based display of clinical pathology laboratory data. Am J Clin Pathol.

[88] Overbeek LI, Hermens RP, Van Krieken JH, Adang EM, Casparie M, Nagengast FM (2010). Electronic reminders for pathologists promote recognition of patients at risk for Lynch syndrome: cluster-randomised controlled trial. Virchows Arch.

[89] Comaniciu D, Meer P, Foran DJ (1999). Image-guided decision support system for pathology. Mach Vis Appl.

[90] Shah SP, Huang Y, Xu T, Yuen MM, Ling J, Ouellette BF (2005). Atlas - a data warehouse for integrative bioinformatics. BMC Bioinformatics.

[91] Lee TJ, Pouliot Y, Wagner V, Gupta P, Stringer-Calvert DW, Tenenbaum JD (2006). BioWarehouse: a bioinformatics database warehouse toolkit. BMC Bioinformatics.

[92] Trissl S, Rother K, Müller H, Steinke T, Koch I, Preissner R (2005). Columba: an integrated database of proteins, structures, and annotations. BMC Bioinformatics.

[93] Choi C, Münch R, Leupold S, Klein J, Siegel I, Thielen B (2007). SYSTOMONAS–an integrated database for systems biology analysis of Pseudomonas. Nucleic Acids Res.

[94] Rhodes DR, Yu J, Shanker K, Deshpande N, Varambally R, Ghosh D (2004). ONCOMINE: a cancer microarray database and integrated data-mining platform. Neoplasia.

[95] Smedley D, Haider S, Ballester B, Holland R, London D, Thorisson G (2009). BioMart–biological queries made easy. BMC Genomics.

[96] Köhler J, Baumbach J, Taubert J, Specht M, Skusa M, Skusa A (2006). Graph-based analysis and visualization of experimental results with Ondex. Bioinformatics.

[97] Smith RN, Aleksic J, Butano D, Carr A, Contrino S, Hu F (2012). InterMine: a flexible data warehouse system for the integration and analysis of heterogeneous biological data. Bioinformatics.

